# Contrast-Induced Neurotoxicity following Cardiac Catheterization

**DOI:** 10.1155/2012/267860

**Published:** 2012-11-06

**Authors:** Susan Law, Kessarin Panichpisal, Melaku Demede, Sabu John, Jonathan D. Marmur, Jaya Nath, Alison E. Baird

**Affiliations:** ^1^Department of Neurology, SUNY Downstate Medical Center, 450 Clarkson Avenue, P.O. Box 1274, Brooklyn, NY 11203, USA; ^2^Department of Neurology, SUNY Downstate Medical Center and Kings County Hospital Center, 450 Clarkson Avenue, Brooklyn, NY 11203, USA; ^3^Division of Neurology, Bumrungrad International Hospital, Bangkok 10110, Thailand; ^4^Divisions of Cardiology, SUNY Downstate Medical Center and Kings County Hospital Center, 450 Clarkson Avenue, Brooklyn, NY 11203, USA; ^5^Divisions of Radiology, SUNY Downstate Medical Center and Kings County Hospital Center, 450 Clarkson Avenue, Brooklyn, NY 11203, USA

## Abstract

We report a case of probable contrast-induced neurotoxicity that followed a technically challenging cardiac catheterization in a 69-year-old woman. The procedure had involved the administration of a large cumulative dose of an iodinated, nonionic contrast medium into the innominate artery: twelve hours following the catheterization, the patient developed a seizure followed by a left hemiplegia, and an initial computed tomography (CT) scan showed sulcal effacement in the right cerebral hemisphere due to cerebral swelling. The patient's clinical symptoms resolved within 24 hours, and magnetic resonance imaging at 32 hours showed resolution of swelling. Contrast-induced neurotoxicity should be found in the differential diagnosis of acute neurological deficits occurring after radiological procedures involving iodinated contrast media, whether ionic or nonionic.

## 1. Introduction

The hazards associated with the contrast agents used for cardiac and cerebral angiography are well known. Aside from allergic reactions and renal toxicity, relatively common neurological complications are vasospasm related to the catheterization, intimal lesions, pseudoaneurysms, and embolism [1]. An uncommon, but important neurological complication, is that of contrast-induced neurotoxicity (CIN) [2].

CIN has been associated with the iodinated contrast media used for intravascular cerebral and cardiac angiography and for intrathecal injections [2]. The mechanism of CIN is unclear. The most likely cause is of osmotic disruption of the blood brain barrier and cerebral edema from the hyperosmolar contrast. Another potential mechanism is that contrast molecules are chemotoxic to neurons. Direct stimulation or excitation of neurons by contrast was seen to result in spontaneous slow waves and electrographic seizures in experimental studies [3–5]. The newest generation of contrast media is nonionic and of lower osmolality than the older ionic contrast media. Nonionic iodinated contrast media have been associated with fewer adverse effects and only rarely with CIN [2, 6].

The clinical presentation of CIN ranges from headache and vomiting to seizures, dysarthria, hemiparesis, hemianopia, parkinsonism, transient global amnesia, and transient cortical blindness [5, 7]. CIN usually appears 2 to 12 hours after the contrast injection and disappears within 24 to 72 hours [7]. Patients with underlying brain conditions, impaired kidney function, those who have received a large contrast dose, and those with prolonged exposure of contrast media are at the greatest risk for developing CIN [5, 7]. Here, we report a case of probable CIN after a technically challenging cardiac catheterization involving intra-arterial injection of a large dose of the nonionic contrast agent iodixanol 320 mgI/mL.

## 2. Case Report

A 69-year-old right-handed woman underwent elective cardiac catheterization because of an abnormal stress test. She had previously undergone coronary artery bypass grafting and also had hypertension, diabetes mellitus, and renal impairment, with an estimated glomerular filtration rate of 49 mL/min/1.73 m^2^.

The cardiac catheterization was performed via a right common femoral artery approach, and injections were made into the left ventricle, the native vessels, and the venous graft—an innominate artery to right coronary artery greater saphenous vein graft. A stent was placed into the venous graft. The procedure was technically difficult and involved repeated injections into the innominate artery. A total of 320 mL of Iodixanol 320 mgI/mL, a dimeric nonionic contrast medium, was administered. The patient was observed to be clinically normal after the procedure. No general anesthetic had been administered during the procedure.

**Figure 1:**
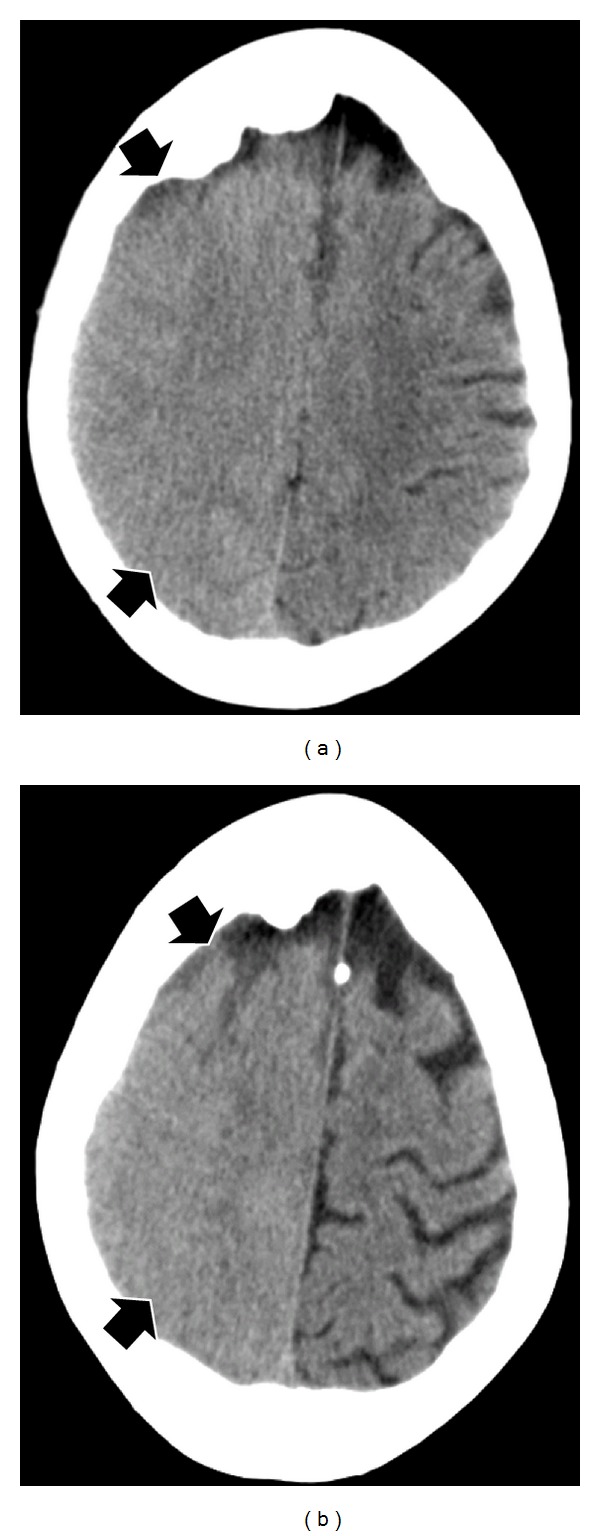
Unenhanced computed tomography obtained one hour after the onset of patient's symptoms ((a) and (b)) showed sulcal effacement in the right cerebral hemisphere due to cerebral swelling. The changes were most marked in the high frontal and parietal lobes.

Twelve hours later, the patient developed a left-sided motor seizure that became secondarily generalized. The seizure was terminated with lorazepam. Postictally, there was left-sided homonymous hemianopia, hemisensory loss, hemiparesis, and hemineglect. The patient was thought to have suffered an ischemic stroke involving the right middle cerebral artery territory. Unenhanced CT scanning performed within 1 hour showed sulcal effacement in the right cerebral hemisphere due to cerebral swelling; the changes were most marked in the high frontal and parietal lobes (Figures 1(a) and 1(b)). There was no hemorrhage or midline shift or evidence of a hyperdense middle cerebral artery sign. The patient was initially assessed as having a stroke. She received intravenous thrombolysis and had returned to her clinical baseline within 24 hours.

**Figure 2:**
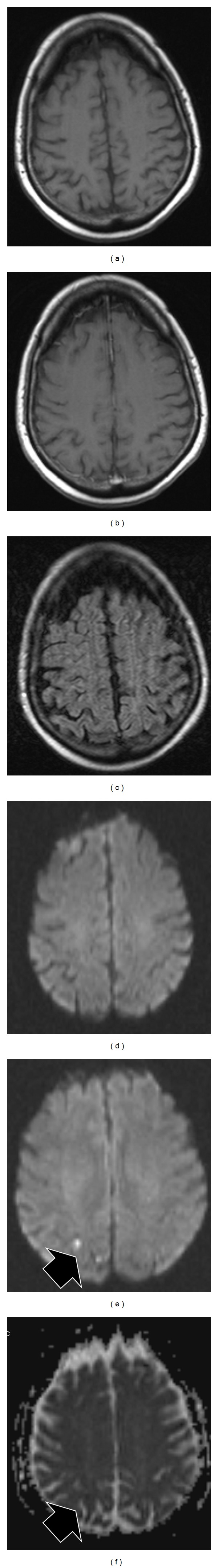
MRI obtained 32 hours after patient's symptoms onset. Top row: T1WI before contrast (a), T1WI after contrast (b), FLAIR-T2 images (c) are unremarkable. On the bottom row, DWI ((d)-(e)) and the corresponding ADC map (f) show two tiny foci of restricted water diffusivity in the right parietal lobe, indicating acute lacunar infarcts.

An MRI at 32 hours from patient's symptoms showed no edema and no swelling or cortical enhancement on the postcontrast T1-weighted images (T1WIs) or on the fluid-attenuated inversion recovery T2-weighted images (FLAIR-T2) (Figures 2(a)–2(c)). Two 1 mm acute infarcts of the right parietal lobe were seen on the diffusion-weighted images (DWIs) (Figures 2(d)–2(f)). A new CT scan at 84 hours confirmed resolution of the hemispheric swelling and absence of bleeding 

It was subsequently determined that one year earlier the patient had experienced transient binocular blurred vision after a cardiac catheterization procedure at an outside hospital. At that time, an unenhanced computed tomography (CT) scan had shown questionable occipital lobe hypodensities that resolved within 24 hours.

## 3. Discussion

The differential diagnoses in this patient included contrast-induced neurotoxicity (CIN), stroke, seizure-induced brain swelling, and subarachnoid hemorrhage. The acute neurological deficits, the initial neuroimaging changes in the symptomatic hemisphere and the correlation of the resolution of the symptoms with the complete resolution of the CT changes made CIN the most likely diagnosis in this patient. The patient had also received a very high dose of iodinated contrast (320 ml of 320 mgI/mL Iodixanol)—the maximal recommended dose being 200 mL—into the symptomatic hemisphere.

The initial clinical diagnosis was of a seizure secondary to acute right hemispheric ischemic stroke in the setting of a technically difficult cardiac catheterization involving the innominate artery. However, the involvement of the whole hemisphere with no definite vascular territory should exclude this diagnosis. Typically, in hyperacute stroke, the brain CT scan is normal or may show subtle areas of hypodensity and/or corticosubcortical blurring that are indicative of early infarction. There may also be a hyperdense middle cerebral artery sign indicative of thrombosis. The rapid improvement of the patient's symptoms also was atypical. The punctate small infarctions on MRI could not explain her significant transient neurological deficits. Another differential diagnosis was that of a first-onset of idiopathic seizure followed by a Todd's paralysis. However, the extent of swelling was too large for seizure-induced brain swelling, and there was a lack of corresponding changes on the MRI [8]. Subarachnoid hemorrhage was also ruled out by the absence of any hyperdensity in the sulci on the initial nonenhanced CT [3, 9, 10]. Hyperperfusion syndrome was unlikely because no carotid revascularization was performed in this patient.

CIN resulting from Iodixanol administration is extremely rare, and this is the second case reported to our knowledge [6]. Iodixanol is an iso-osmolar contrast agent with an osmolarity similar to that of blood [11]. Theoretically, iso-osmolar contrast agents are expected to be safer than noniso-osmolar ones, since they hardly induce blood brain barrier damage. We speculate that patient's blood brain barrier in the right hemisphere might impaired by small ischemic strokes from microemboli occurring during the cardiac catheterization or from her underlying carotid stenosis. Additionally, she had received a very high dose of contrast and had baseline impaired kidney function [12], that likely resulted in an unusually high concentration of contrast to the brain with subsequent neurotoxicity: a large dose of contrast (regardless of osmolality) itself can cause disruption of the blood brain barrier and neurotoxicity in the absence of underlying brain pathology [12, 13]. Finally, the patient had a previous history of transient cortical blindness after cardiac catheterization which may also be associated to CIN as well. 

It is important to keep in mind that various radiographic findings have been associated with CIN [5]. Initial CT scans may be normal, may demonstrate cortical and/or subcortical enhancement, cerebral edema, and/or hyperdensity in the subarachnoid space or parenchyma mimicking subarachnoid hemorrhage, or intracerebral hemorrhage [5, 9, 10, 14]. Measuring the Hounsfield units (HUs) in doubtful cases can assist in differentiating blood from contrast as contrast media present higher attenuation (80–160 HU) than blood (40 to 60 HU) [5]. 

The prognosis of CIN is excellent. Although there is no specific treatment for this condition, steroids and mannitol have been administrated in some cases [1]. Seizures are easily controlled with benzodiazepines [12]. Since the safety of future contrast exposure in patients with this type of reaction has not been studied extensively, extreme caution should be exercised when a repeat study with contrast is considered.

In conclusion, CIN is a rare condition that should be taken into account in the differential diagnoses of acute focal or generalized neurological symptoms after procedures involving the use of intracarotid injection of iodinated contrast media, whether ionic or nonionic. Its recognition may be crucial for patient management. This syndrome can be differentiated from stroke on the basis of clinical and imaging findings.
